# Delayed neointimal healing pattern after bioresorbable scaffold implantation

**DOI:** 10.1007/s12471-017-1070-4

**Published:** 2018-01-02

**Authors:** D. Ochijewicz, M. Tomaniak, J. Kochman, L. Koltowski, A. Rdzanek, A. Pietrasik, G. Opolski

**Affiliations:** First Chair and Department of Cardiology, Subdivision of Interventional Cardiology, Medical University of Warsaw, Warsaw, Poland

A 67-year-old male underwent primary percutaneous coronary intervention for inferior ST-segment elevation myocardial infarction. The patient further underwent aspiration thrombectomy, abciximab infusion and predilatation with subsequent implantation of two bioresorbable scaffolds followed by post-dilatation with a non-compliant balloon in the right coronary artery. The optical coherence tomography (OCT) confirmed good scaffold expansion, with focally malapposed struts in the distal 3‑mm scaffold segment. Twelve months later, at the prespecified evaluation, the vessel was patent without evidence of restenosis or thrombus, however, the OCT revealed a substantial rate of uncovered struts (8.5%). Interestingly, all struts were covered by neointima at the 24-month examination. Based on these findings, prolonged dual antiplatelet therapy was ceased after two years from the index procedure. Discontinuation of P2Y12 antagonist at 12 months potentially could have led to late scaffold thrombosis in our patient with initially impaired endothelial healing response (see Fig. 1).

**Fig. 1:**
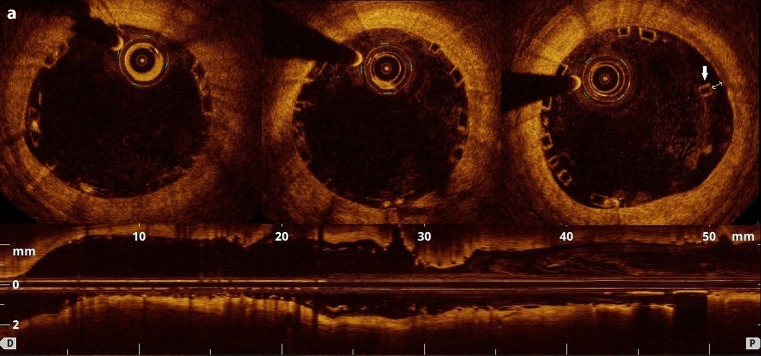
**a** (Baseline) Optical coherence tomography (OCT) directly after implantation of the bioresorbable scaffold (BVS). Adequate BVS expansion and focally malapposed struts in the distal 3‑mm scaffold segment (*white arrow*) into right coronary artery. **b** (1-year follow-up) Uncovered stent struts (*blue arrows*) at 12 months after the intervention. **c** (2-year follow-up) 24-month OCT revealed complete coverage of all struts with a homogeneous, bright neointimal layer and resolved malapposition in the distal segment

**Fig. 1:**
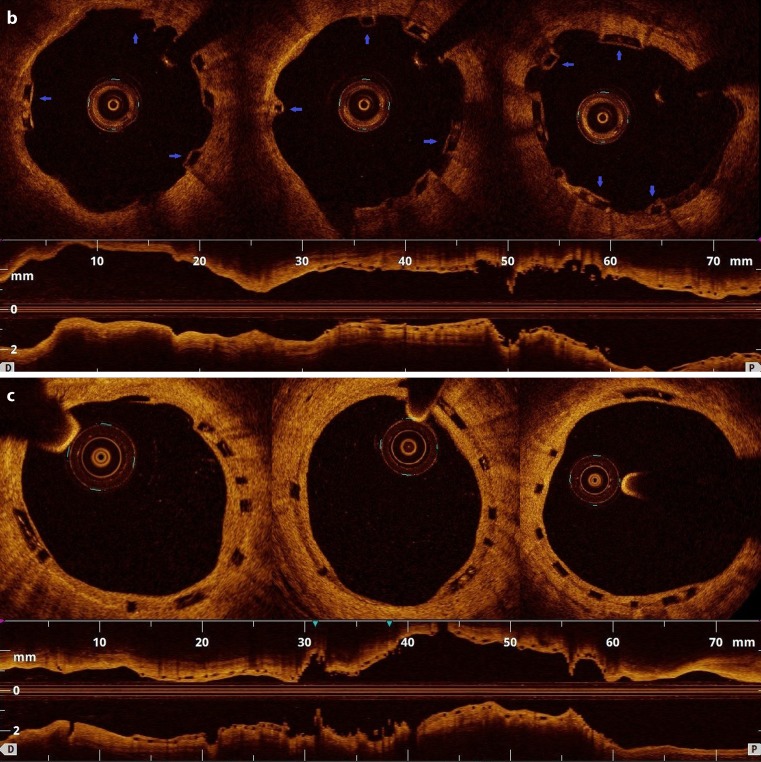
(continued)

